# A Novel Mutation in *LEPRE1* That Eliminates Only the KDEL ER- Retrieval Sequence Causes Non-Lethal Osteogenesis Imperfecta

**DOI:** 10.1371/journal.pone.0036809

**Published:** 2012-05-15

**Authors:** Masaki Takagi, Tomohiro Ishii, Aileen M. Barnes, MaryAnn Weis, Naoko Amano, Mamoru Tanaka, Ryuji Fukuzawa, Gen Nishimura, David R. Eyre, Joan C. Marini, Tomonobu Hasegawa

**Affiliations:** 1 Department of Pediatrics, Keio University School of Medicine, Tokyo, Japan; 2 Department of Endocrinology and Metabolism, Tokyo Metropolitan Children's Medical Center, Tokyo, Japan; 3 Bone and Extracellular Matrix Branch, NICHD, NIH, Bethesda, Maryland, United States of America; 4 Orthopaedic Research Laboratories, University of Washington, Seattle, Washington, United States of America; 5 Department of Obstetrics and Gynecology, Keio University School of Medicine, Tokyo, Japan; 6 Department of Pathology and Laboratory Medicine, Tokyo Metropolitan Children's Medical Center, Tokyo, Japan; 7 Department of Radiology, Tokyo Metropolitan Children's Medical Center, Tokyo, Japan; Russian Academy of Sciences, Institute for Biological Instrumentation, Russian Federation

## Abstract

Prolyl 3-hydroxylase 1 (P3H1), encoded by the *LEPRE1* gene, forms a molecular complex with cartilage-associated protein (CRTAP) and cyclophilin B (encoded by *PPIB*) in the endoplasmic reticulum (ER). This complex is responsible for one step in collagen post-translational modification, the prolyl 3-hydroxylation of specific proline residues, specifically α1(I) Pro986. P3H1 provides the enzymatic activity of the complex and has a Lys-Asp-Glu-Leu (KDEL) ER-retrieval sequence at the carboxyl terminus. Loss of function mutations in *LEPRE1* lead to the Pro986 residue remaining unmodified and lead to slow folding and excessive helical post-translational modification of type I collagen, which is seen in both dominant and recessive osteogenesis imperfecta (OI). Here, we present the case of siblings with non-lethal OI due to novel compound heterozygous mutations in *LEPRE1* (c.484delG and c.2155dupC). The results of RNA analysis and real-time PCR suggest that mRNA with c.2155dupC escapes from nonsense-mediated RNA decay. Without the KDEL ER- retrieval sequence, the product of the c.2155dupC variant cannot be retained in the ER. This is the first report of a mutation in *LEPRE1* that eliminates only the KDEL ER-retrieval sequence, whereas other functional domains remain intact. Our study shows, for the first time, that the KDEL ER- retrieval sequence is essential for P3H1 functionality and that a defect in KDEL is sufficient for disease onset.

## Introduction

Osteogenesis imperfecta (OI; MIM #166200, #166210, #259420, #166220, #610967, #610968, #610682, #610915, #259440, #613848 and #613982) comprises a heterogeneous group of connective tissue disorders characterized by fragile bones with susceptibility to fractures. Most cases of OI are caused by heterozygous mutations in *COL1A1* or *COL1A2*, the genes encoding the two type I procollagen alpha chains, proα1 (I) and proα2 (I) [1]. Mutations in these genes result in quantitative and/or qualitative defects in type I collagen production by osteoblasts [2]–[4].

Recurrence of severe OI in families with unaffected parents results from either dominant (parental mosaicism) or recessive inheritance [5]–[7]. Recent investigations have discovered several genes responsible for OI inherited as an autosomal recessive trait [8]–[18]. Among these genes, *LEPRE1* encodes prolyl 3-hydroxylase 1 (P3H1), which forms a molecular complex with cartilage-associated protein (CRTAP) and cyclophilin B (CypB, encoded by *PPIB*) in the endoplasmic reticulum (ER) that is responsible for one step in collagen post-translational modification, the prolyl 3-hydroxylation of specific proline residues, specifically α1(I) Pro986 [19]. P3H1 provides the enzymatic activity of the complex and is the only component of the complex with a Lys-Asp-Glu-Leu (KDEL) ER-retrieval sequence at the carboxyl terminus [20]. Loss of function mutations in either *LEPRE1* or *CRTAP* lead to loss of both proteins in the cell, leave the Pro986 residue unmodified, and lead to slow folding and excessive helical post-translational modification of type I collagen [21].

To date, more than 20 *LEPRE1* mutations have been described [10], [21]–[25]. With the exception of only one missense mutation, Leu489Pro [25], all *LEPRE1* mutations result in a premature termination codon (PTC) with mRNA that is destroyed by the process of nonsense-mediated RNA decay. Here we present the case of siblings with OI due to novel compound heterozygous mutations in *LEPRE1* (c.484delG and c.2155dupC). Without the KDEL ER- retrieval sequence, the product of the c.2155dupC variant cannot be retained in the ER. Our study shows, for the first time, that the KDEL ER- retrieval sequence is essential for P3H1 functionality and that a defect in KDEL is sufficient for disease onset.

## Results

### Patient Reports

**Figure 1:**
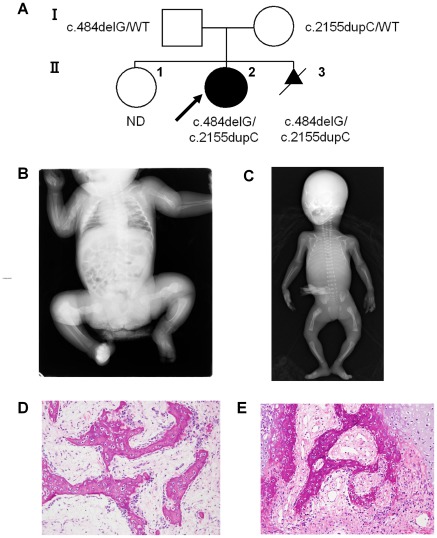
Features of Siblings with Mutations of *LEPRE1.* A: The pedigree of the affected family. The arrow indicates the proband. Patient II-3 was electively terminated. B: Radiographs of Patient II-2 as a neonate. There were multiple rib fractures, healed fractures of both femora and the right humerus, and a subacute fracture of the left humerus. Metaphyseal osteopenia was significant. C: Postmortem radiographs of Patient II-3. Bilateral femoral bowing, a healed fracture of the right femoral shaft, thin ribs, and metaphyseal demineralization were shown. D, E: Histological findings of Patient II-3. Irregular trabeculae of woven bones lined by osteoblasts are observed in the humerus (D) and spine (E). The stroma is cellular and consists of fibroblasts and collagen resembling osteofibrous dysplasia.

Patient II-2 was a 5-year-old female born to healthy parents who already had one healthy child (Fig 1A). Prenatal ultrasonography at 28 weeks of gestation showed deformity of the lower limbs. She was delivered with multiple fractures by caesarian section at 35 weeks’ gestation. Birth weight was 1966 g (below 3^rd^ percentile), length 42.2 cm (below 3^rd^ percentile), and OFC 31.2 cm (3^rd^–10^th^ percentile). She did not have blue sclera or dysmorphic facial features, such as micrognathia or a triangular face. She had no neonatal respiratory distress. Radiographs showed multiple rib fractures, healed fractures of both femora and the right humerus, and a subacute fracture of the left humerus (Fig 1B). Metaphyseal osteopenia was significant. A diagnosis of OI type III was made. At least 10 fractures occurred in the first 6 months of life. Pamidronate treatment was initiated at 2 months of age. The pamidronate was initially administered by infusion every 2 months and was changed to every 3 months at the age of 2 years. The bone mineral density (BMD) of the lumber spine (L2–L4) was 0.336 gm/cm^2^ (Z score of –2.2), 0.429 g/cm^2^ (Z score of –2.7), 0.479 g/cm^2^ (Z score of –4.9), and 0.514 g/cm^2^ (Z score of –5.9) at the ages of 1 year, 2 years, 4 years, and 5 years respectively (We used BMD reference data [26] in Spanish children). She did not have severe deformity of the long bones at age 5 years, and her skin was normal in extensibility. She had white sclerae and normal dentition. She was able to walk with difficulty while holding on to a table. Her intellectual development was normal.

Patient II-3 was the product of couple's next pregnancy; this pregnancy was electively terminated. Postmortem radiographs showed bilateral femoral bowing, a healed fracture of the right femoral shaft, thin ribs, and metaphyseal demineralization (Fig 1C).

### Patient II-3 Bone Histology

Bone samples, obtained at autopsy, from Patient II-3 were processed according to standard procedure, and the formalin fixed paraffin-embedded sections were stained with hematoxylin and eosin. Irregular trabeculae of woven bone rimed by osteoblasts were observed in the humerus (Fig 1D) and spine (Fig 1E). The stroma surrounding the woven bone was mildly to moderately cellular and consisted of fibroblasts and collagen. These histological features resembled those of osteofibrous dysplasia.

### Detection of *LEPRE1* Mutations

**Figure 2:**
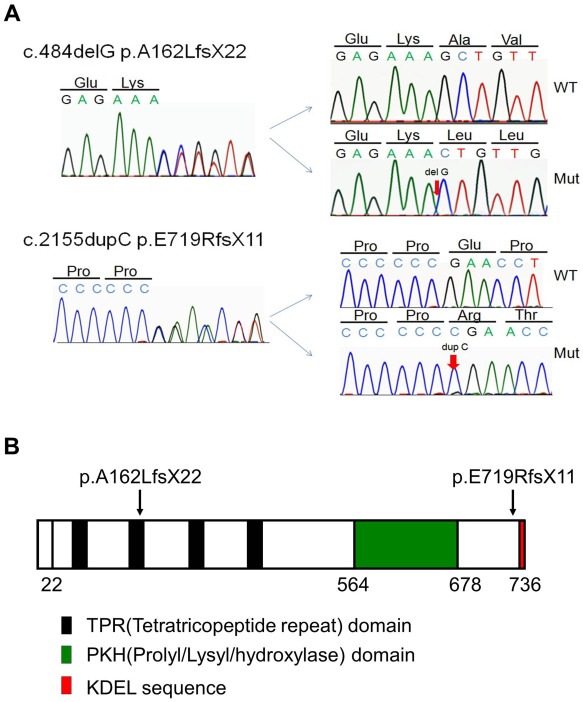
Identification of *LEPRE1* mutations. A: A partial sequence of PCR product of Patient II-3 is shown. Compound heterozygous frame shift mutations (c.484delG, p.A162LfsX22 and c.2155dupC, p.E719RfsX11) are indicated by arrows. The mutations have been confirmed by the subsequent sequencing of subcloned products of normal and mutant alleles. B: Schematic presentation of the positions of the mutation. *LEPRE1* cDNA encodes the tetratricopeptide repeat domain (four black regions), the Prolyl/Lysyl/hydroxylase domain (green region), and the KDEL ER- retrieval motif (red region). *LEPRE1* with a p.E719RfsX11 change results in the lack of only the KDEL ER-retrieval sequence, whereas other functional domains remain intact.

Sequence analysis revealed novel compound heterozygous *LEPRE1* mutations (c.484delG, p.A162LfsX22 and c.2155dupC, p.E719RfsX11) in both patients (Fig 2A). Their father carried c.484delG and their mother carried c.2155dupC. These mutations were not found in 200 control alleles. No sequence variation was found in *COL1A1, COL1A2*, *CRTAP*, or *PPIB*, and neither exon-level deletion nor duplication involving *COL1A1* and *COL1A2* was detected by MLPA analysis. The p.E719RfsX11 mutation creates a PTC in the last exon and results in the lack of only the KDEL ER-retrieval sequence, whereas other functional domains, such as the tetratricopeptide domain and Prolyl/Lysyl hydroxylase domain, remain intact (Fig 2B).

### *LEPRE1* Transcripts and P3H1 Protein in Probands

Only the allele with c.2155 dupC was successfully amplified and sequenced at the cDNA level.

**Figure 3:**
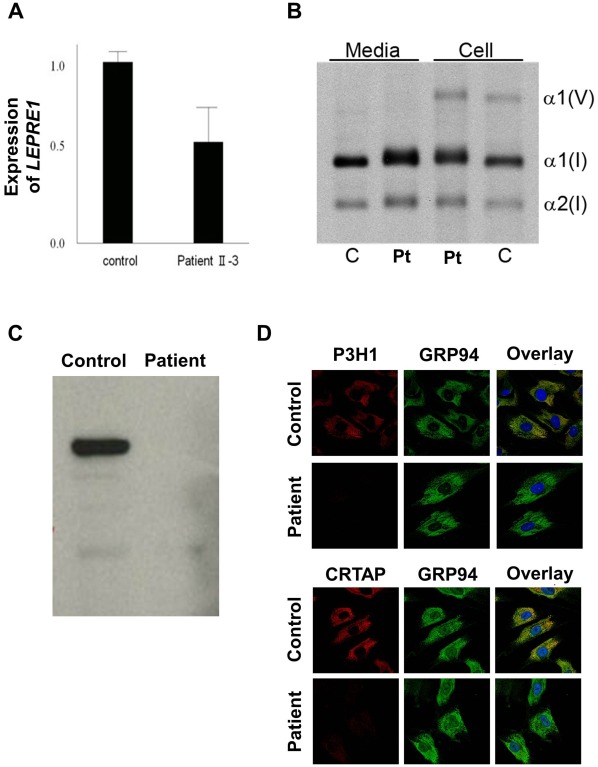
Characterization of the *LEPRE1* mutations and proband collagen. A: Patient II-3 *LEPRE1* transcripts are about one-half the control level, by real-time RT-PCR. B: Steady-state type I collagen protein from fibroblasts of Patient II-3 and a normal control is shown. In both the cell layer and media, overmodification, detected as back-streaking of collagen alpha chains (α1 (I) and α2 (I)) on gel electrophoresis, is present in Patient II-3. We also detected mild overmodification of type V collagen (α1 (V)). C: Western blots of fibroblast P3H1 in Patient II-3 and control cells confirm absence of intracellular P3H1. D: Immunofluorescent staining of fibroblasts from Patient II-3 and a normal control show colocalization of P3H1 and CRTAP with GRP94 in control cells. Both P3H1 and CRTAP proteins are absent in fibroblasts from Patient II-3.

Real-Time PCR revealed that the level of *LEPRE1* transcripts of Patient II-3 was about one-half the control level (Fig 3A).

Western blot analysis of fibroblast lysates confirmed the absence of intracellular P3H1 in Patient II-3 (Fig 3C). Fluorescent microscopy showed the expected colocalization of P3H1 and CRTAP with GRP94 in control cells. Both P3H1 and CRTAP proteins were absent in fibroblasts from Patient II-3 (Fig 3D), reflecting mutual protection in the complex.

### Collagen Post-translational Modification

In both the cell layer and media, steady-state fibroblast collagen of Patient II-3 displayed helical overmodification, detected as back-streaking of collagen alpha chain bands on gel electrophoresis (Fig 3B).

Tandem mass spectrometry analysis of tryptic peptides of Patient II-3 secreted α1 (I)-collagen chains revealed only a slight reduction (85% in proband, 95–98% in control collagen) of Pro986 3-hydroxylation (data not shown) despite the absence of detectable mutant P3H1 protein in the cell.

## Discussion

ER-resident proteins must be distinguished from newly synthesized secretory proteins, which pass through this compartment as they transit the secretory pathway toward the extracellular space. One of the mechanisms by which this is achieved is the selective retrograde transport of soluble ER-resident proteins from the cis-Golgi to the ER [27]. Receptors in post-ER compartments recognize a C-terminal motif that marks proteins that are to be retained in the ER. The KDEL motif binds to this salvaging receptor (KDEL receptor) in the Golgi, resulting in this ligand-receptor complex being returned to the ER [27]. Soluble ER-resident proteins such as molecular chaperones and components of the control quality machinery, e.g. immunoglobulin heavy-chain binding protein, calreticulin, and protein disulfide isomerase, contain the KDEL motif at the carboxyl terminus. P3H1, encoded by *LEPRE1*, forms a molecular complex with CRTAP and CypB in the ER, and provides the enzymatic activity of the complex. P3H1 is the only component of the complex with a KDEL ER-retrieval sequence at the carboxyl terminus [20]. One splice mutation, c.2055+18G>A, which abolishes the *LEPRE1* mRNA splice form of KDEL, has previously been reported [23]. This splice mutation results in preferential use of alternative splice donor site, and a significant decrease in the *LEPRE1* mRNA splice form containing the KDEL sequence. However, this finding does not provide direct evidence for the importance of the KDEL sequence. The case presented here is therefore the first report of a mutation in *LEPRE1* that eliminates only the KDEL ER-retrieval sequence, while all other functional domains remain intact. Without the KDEL ER- retrieval sequence, the c.2155dupC variant will not captured by KDEL receptor in the Golgi. Our report shows, for the first time, that the KDEL ER- retrieval sequence is essential for P3H1 functionality *in vivo*. Dysfunction of this KDEL-KDEL receptor interaction will provide us one disease causing mechanism of OI as well as other diseases involved in ER enzyme.

It is noteworthy that our proband’s collagen contained higher percentage (85%) of 3-hydroxylated Pro986 residues than previously reported with *LEPRE1* null mutations, which showed severely reduced (0–15%) 3-hydroxylation of Pro986 [10], [22], [23]. We could not detect mutant P3H1 in the proband cells by western blotting assay or fluorescent microscopy. However, we hypothesize that the P3H1/CRTAP/CyPB complex that includes the mutant P3H1 without KDEL must be transiently present in the ER at some minimal level, which is sufficient for 3-hydroxylating most α1(I) Pro986 residues. Recently, it was reported that the P3H1/CRTAP/CyPB complex has 3 distinct activities: it is a prolyl 3-hydroxylase, a PPIase, and a molecular chaperone [28]. In the present patient, despite the higher percentage of 3-hydroxylated Pro986 residues, overmodification of the patient’s type I collagen was observed electrophoretically. This observation implicates the dysfunctional P3H1/CRTAP/CyPB complex in the pathology, with potential roles for absence of its chaperone or PPIase functions. However, since our proband has generally milder OI than described for null *LEPRE1* mutations, the OI severity may correlate with the level of type I collagen P986 3-hydroxylation.

In conclusion, our study shows, for the first time, that the KDEL ER- retrieval sequence is important for P3H1 functionality *in vivo*. In addition, the higher percentage of 3-hydroxylated P986 residues seen in the collagen of our patient correlates with her moderate phenotype, in contrast to the severe/lethal OI of probands with null *LEPRE1* mutations and minimal collagen 3-hydroxylation.

## Materials and Methods

### PCR-Based Mutation Screening

Approval for this study was obtained from the Institutional Review Board of Keio University School of Medicine. The parents gave written informed consent for the molecular studies.

Genomic DNA was extracted from peripheral blood (Patient II-2) and blood of the umbilical cord (Patient II-3) by a standard technique. We analyzed all coding exons and flanking introns of *COL1A1, COL1A2, LEPRE1, CRTAP,* and *PPIB* by PCR and direct sequencing. Deletion or duplication involving *COL1A1* and *COL1A2* was checked by multiplex ligation-dependent probe amplification (MLPA) analyses (SALSA MLPA KIT P271, P272; MRC-Holland, Amsterdam, The Netherlands).

### RNA Analysis and Real-Time PCR

Total RNA was extracted from skin fibroblasts of Patient II-3 and cDNA synthesis was performed with the SuperScript III reverse transcriptase kit (Invitrogen, Carlsbad, CA) with oligoDT primers. Exons 2 and 15 of *LEPRE1* were amplified from cDNA by PCR. Subsequently, the PCR products were subjected to direct sequencing.

Real-time quantitative PCR was performed on the ABI PRISM 7500 Fast Real-Time PCR System (Applied Biosystems, Foster City, CA). For PCR reaction, we used SYBR Premix Ex Taq II (Takara, Otsu, Japan). *LEPRE1* expression was calculated using a control fibroblast mRNA standard curve, then normalized to a constitutively expressed gene (b2-microglobulin). All reactions were carried out in triplicate and expression levels were determined in 3 independent experiments.

### Western Blotting

Skin fibroblasts from Patient II-3 and a control subject were cultured in Dulbecco's modified Eagle's medium (DMEM) and were lysed in RIPA buffer (Sigma). Samples were subjected to 10% SDS-PAGE and then transferred onto polyvinylidene fluoride membrane. The membrane was treated with 10% milk powder solution overnight at 4°C, and incubated with primary antibody: mouse anti-LEPRE1 MaxPab polyclonal antibody (Abnova, Taipei, Taiwan) at a 1∶1000 dilution. After washing, the membrane was incubated with secondary antibody: goat anti-mouse HRP conjugated (Invitrogen) at a 1∶1000 dilution. The membrane was washed again and then scanned to visualize the specific protein band.

### Steady-state Collagen Analysis

Control and Patient II-3 dermal fibroblasts were grown to confluence in DMEM + Glutamax™ supplemented with 10% fetal bovine serum and penicillin/streptomycin. Cells were labeled overnight in serum-free medium containing 50 µg/ml ascorbic acid and 437.5 µCi/ml L-[2,3,4,5-^3^H]proline. Collagens were precipitated with ammonium sulfate, pepsin-digested and separated on 6% SDS-Urea PAGE.

### Immunocytochemistry

Immunofluorescence microscopy was performed as described [21]. Control and Patient II-3 dermal fibroblasts were grown on chamber slides. For CRTAP/GRP94 staining, cells were fixed in 4% paraformaldehyde, permeabilized with 0.1% TritonX-100 on ice, and blocked in 1% BSA in PBS. Cells were then incubated overnight with primary antibody (CRTAP, Abnova, Taipei, Taiwan; GRP94, Abcam, Cambridge, MA). After washing, cells were incubated with 1∶200 Alexa Fluor secondary antibodies (Invitrogen) in blocking buffer for 1 h, washed, and mounted with coverslips. Cells were imaged using a Zeiss LSM 510 Inverted Meta microscope and LSM510 software. P3H1/GRP94 staining was done following the protocol of Willaert *et al* [23]. Cells were washed, then fixed and permeabilized in cold acetone. Cells were then blocked in 10% goat serum and incubated with primary antibody (LEPRE1 MaxPab, Abnova, Taipei, Taiwan) for 2.5 h. Secondary staining and imaging was done as above.

### Tandem Mass Spectrometry

Secreted collagens from ascorbic acid stimulated fibroblast cultures were precipitated and the α1(I) bands were isolated and digested with trypsin. Electrospray mass spectrometry was performed as before [9].
